# Linking the YTH domain to cancer: the importance of YTH family proteins in epigenetics

**DOI:** 10.1038/s41419-021-03625-8

**Published:** 2021-04-01

**Authors:** Rongkai Shi, Shilong Ying, Yadan Li, Liyuan Zhu, Xian Wang, Hongchuan Jin

**Affiliations:** 1 Laboratory of Cancer Biology, Key Lab of Biotherapy in Zhejiang, Sir Run Run Shaw Hospital, Cancer Center, Zhejiang University, Hangzhou, China; 2 Department of Medical Oncology, Sir Run Run Shaw Hospital, Medical School of Zhejiang University, Hangzhou, China

**Keywords:** Cancer, Epigenetics

## Abstract

N6-methyladenosine (m6A), the most prevalent and reversible modification of mRNA in mammalian cells, has recently been extensively studied in epigenetic regulation. YTH family proteins, whose YTH domain can recognize and bind m6A-containing RNA, are the main “readers” of m6A modification. YTH family proteins perform different functions to determine the metabolic fate of m6A-modified RNA. The crystal structure of the YTH domain has been completely resolved, highlighting the important roles of several conserved residues of the YTH domain in the specific recognition of m6A-modified RNAs. Upstream and downstream targets have been successively revealed in different cancer types and the role of YTH family proteins has been emphasized in m6A research. This review describes the regulation of RNAs by YTH family proteins, the structural features of the YTH domain, and the connections of YTH family proteins with human cancers.

## Facts

- The effects of YTH proteins on RNA metabolism are different but overlap to some degree.
- The structure of the YTH domain helps YTHDF proteins recognize and bind m6A-containing transcripts.
- Structural crystallography studies have elaborated the molecular basis of YTH domains to read m6A-modified RNA.
- YTH proteins have different targets in different cancers and are involved in almost every aspect of tumorigenesis and cancer progression.

## Open questions

- What is the precise unified model of YTH proteins in the regulation of m6A modification? Are these proteins different or redundant?
- Is evaluating structural differences in YTH domains the potential direction for exploring and explaining the complex phenomena in this field?
- Is a highly selective YTH domain inhibitor a potential therapeutic agent for cancer?
- Is the existing structural information about the YTH domain useful for guiding the rational design of selective YTH domain inhibitors?

## Introduction

Since the 1950s, nucleotides, which are the basic molecular components of RNA, have been found to undergo a number of chemical modifications on their adenosine (A), guanosine (G), cytidine (C), and uridine (U) nucleosides. Over 100 kinds of RNA modifications, such as hm5C, m1A, and m6A, have been found and these modifications can affect the biogenesis, structure, and function of RNA in different ways; these modifications have been the hotspots in epigenetics in recent years and still have great potential for exploration. N6-methyladenosine (m6A) has been considered the most prevalent and reversible modification of mRNA in eukaryotic cells since its initial discovery in the 1970s^1^. m6A modification is generally located in the consensus motif DRACH (D = G, A, or U; R = G or A; H = A, C, or U), which is enriched in the coding sequence (CDS) and 3′ untranslated region (3′ UTR) of RNA^1–4^. The regulation of m6A depends on three important factors, “writers”, “erasers”, and “readers”. “Writers” and “erasers” add and remove m6A modifications to and from RNA, respectively, while “readers” recognize m6A and affect the fate of RNA. Generally, m6A reader proteins can be divided into three classes, which use the YTH domain (YTH family proteins), m6A switch mechanism (hnRNPC, hnRNPG, and hnRNPA2B1), or common RNA-binding domain and its flanking regions (IGF2BPs and hnRNPA2B1) to bind m6A-containing transcripts^5^. In recent years, writers and erasers have been actively researched^6^, and recently, the focus has gradually turned to readers, especially YTH family proteins, due to the application of several novel methods. m6A-seq, based on antibody-mediated capture and massively parallel sequencing, helps us identify m6A sites at the transcriptome level. In addition to traditional methods such as RIP-seq, new techniques such as CLIP-seq and PAR-CLIP, based on combining immunoprecipitation with in vivo UV crosslinking enhanced by photoactivatable ribonucleosides, help us identify the targets of m6A readers^7–10^. These methods are shedding new light on RNA modifications. Therefore, we attempt to review the regulation of transcripts by YTH family proteins, the structural basis of the YTH domain, and the association of YTH family proteins with human cancers.

## YTH family proteins recognize m6A and regulate RNA processes

**Fig. 1:**
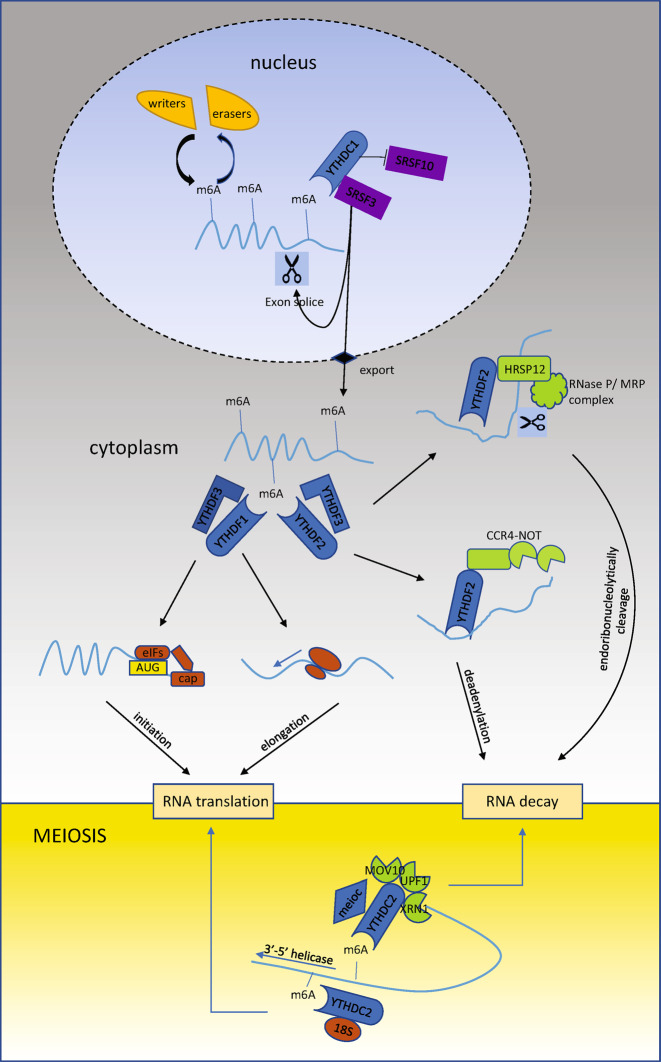
Model of YTH family proteins modulating m6A-containing RNAs. In cell nucleus, “writers” and “erasers” add and remove m6A modifications to and from RNA. YTHDC1 regulates splicing and mediates the export of m6A-containing mRNAs by recruiting SRSF3, while blocking SRSF10 mRNA binding. In cytoplasm, YTHDF1 recognizes m6A-containing mRNAs and promotes its translation initiation and elongation. And the m6A-containing mRNAs can also be recognized by YTHDF2, which promotes their degradation through two pathways, CCR4-NOT complex-mediated deadenylation and HRSP12-mediated endoribonucleolytic cleavage. YTHDF3 interacts with YTHDF1 and YTHDF2 to accelerate metabolism of m6A-modified mRNAs. YTHDC2 mainly functions to regulate the switch from mitosis to meiosis by interacting with MEIOC. YTHDC2 interacts with XRN1, UPF1, and MOV10 to destabilize its target RNAs. Also, the binding of YTHDC2 to the 18S rRNA and its 3′-5′ RNA helicase activity facilitates the translation of its target RNAs.

m6A modification is regulated by RNA methyltransferase complexes—writers—and demethylases—erasers. To catalyze N6-methyladenosine (m6A) RNA methylation, methyltransferase-like 3 (METTL3) and human methyltransferase-like 14 (METTL14) form a stable heterodimer core complex with Wilms tumor 1-associated protein (WTAP), which enables the complex to localize to nuclear speckles enriched with pre-mRNA processing factors^11,12^. Additionally, other adaptor proteins, such as RBM15/15B, VIRMA, and ZC3H13, have been shown to be important for facilitating the function of the methyltransferase complex^13–15^. Regarding demethylases, only fat mass and obesity-associated (FTO) and AlkB homolog 5 (ALKBH5) have been found to be available to catalyze m6A demethylation thus far^16,17^. The YTH domain, the structural basis for the recognition and binding of m6A-modified RNA, enables a series of proteins such as YTHDF1-3 and YTHDC1-2 to act as readers in the regulation of m6A-containing transcripts. Different YTH family proteins function in different ways to influence RNA splicing, export, translation, and decay (Fig. 1).

### YTHDF1-3

YTHDF1 selectively recognizes m6A-modified mRNAs via the YTH domain, promotes their loading into the ribosome, and interacts with initiation factors to facilitate their translation via the N-terminal domain^18^. In addition to mediating translation initiation, YTHDF1 can also bind to the m6A site in the CDS of some mRNAs to assist with translation elongation^19^. Additionally, some research has indicated that YTHDF1 may sometimes mediate the target transcript’s stability^20–22^. It is worth mentioning that YTHDF1 is of great importance in the field of neuroscience since it was reported to be capable of inducing axon regeneration^23^, regulating axon guidance^24^, and thereby facilitating learning and memory^25^ by enhancing the translation of specific transcripts. Conversely, YTHDF2 selectively binds m6A-modified RNA and regulates its degradation by recruiting the CCR4-NOT complex to accelerate RNA deadenylation^10,26,27^. Another YTHDF2-mediated RNA degradation mechanism is endoribonucleolytic cleavage via HRSP12-mediated bridging of YTHDF2-bound RNAs to RNase P/MRP, through which a subset of circular RNAs is selectively downregulated in an m6A-dependent manner^28^. The P/Q/N-rich N-terminal domain of YTHDF2 is responsible for its function in RNA decay, while its aa 101–200 region interacts with the SH domain of CNOT1, and its aa 1–100 region is the HRSP12 binding region. In addition, YTHDF3 interacts with YTHDF1 to help promote protein translation, and with YTHDF2 to affect the decay of methylated mRNA transcripts^29^. There is also a model of m6A regulation in which pre-mRNA is first recognized by YTHDF3, which acts as an assigner, and YTHDF1 and YTHDF2 then competitively interact with YTHDF3, thus determining the fate of the mRNA transcript^30^. Importantly, recent studies have revealed that YTHDFs can be involved in liquid-liquid phase separation by binding multi-m6A-modified mRNA scaffolds and then forming YTHDF–m6A–mRNA complexes. These complexes then partition into phase-separated compartments such as P bodies, stress granules, or neuronal RNA granules to decide whether the mRNA should be degraded, translated, or undergo other events. Especially in stress granule formation, YTHDF proteins are critical in recruiting mRNA to stress granules. In addition, this phase separation is significantly enhanced by mRNAs that contain multiple m6A motifs. In contrast to mRNAs containing a single m6A site, mRNAs containing multiple m6A sites tend to act as scaffolds for the binding of YTHDF proteins to juxtapose their low-complexity domains and initiate phase separation^31–34^. Remarkably, given that YTH domain-containing proteins are m6A readers, it has also been reported that YTHDF2 and YTHDF3 can influence m6A modification levels by blocking the demethylase activity of FTO and ALKBH in different ways, for instance, in heat shock stress-induced transcripts^35–37^. However, recently, some researchers developed a fundamentally different model to explain the effect of YTHDF proteins on m6A-containing transcripts. They hypothesized that the function of YTHDF proteins is redundant and that each paralog compensates for the function of the other paralogs. More importantly, YTHDF proteins show identical binding to all m6A-containing transcripts and function together to promote the decay of these transcripts^38–40^.

### YTHDC1-2

YTHDC1, also called YT521-B, was first found to be an RNA splicing-related protein in 1988 because of its glutamic acid/arginine-rich carboxy domain with splicing factors^41^. It is a ubiquitously expressed nuclear protein and localized in YT bodies adjacent to nuclear speckles^42^. It interacts with SAF-B and Sam68 to modulate alternative splice site selection in a concentration-dependent manner, which is regulated by tyrosine phosphorylation mediated by src kinases^43^. Additionally, YTHDC1 shuttles between the nucleus and the cytosol, where it is phosphorylated by SRC and TEC tyrosine kinase family members, causing its transformed function in RNA splicing^44^. Recently, as the ability of the YTH domain to recognize m6A has gradually been recognized, the association between YTHDC1 and m6A has been partially clarified. YTHDC1 promotes exon inclusion in m6A-modified RNA transcripts in an m6A-dependent manner by recruiting the pre-mRNA splicing factor SRSF3 while blocking SRSF10 mRNA binding to regulate splicing^45^. The interaction with SRSF3 helps YTHDC1 steer target mRNAs into the mRNA nuclear export pathway; thus, YTHDC1 mediates the export of m6A-containing mRNAs^46^. For instance, YTHDC1 mediates m6A-dependent mRNA splicing to control neuronal functions and fine-tune sex determination in Drosophila^47,48^. In addition to playing roles in mRNA splicing and export, YTHDC1 was recently found to regulate the stability of mRNA. YTHDC1 facilitates the decay of chromosome-associated regulatory RNAs in an m6A-dependent manner through nuclear exosome targeting-mediated nuclear degradation and thus decreases chromatin accessibility and represses gene expression^49^. Furthermore, YTHDC1 is involved in controlling the stability of MAT2A mRNA, which is upregulated through mRNA stabilization in response to *S*-adenosylmethionine depletion^50^. Importantly, YTHDC1 recognizes m6A residues on XIST, which is essential for XIST’s function in mediating the transcriptional silencing of genes on the X chromosome^13^. However, the mechanism by which YTHDC1 binding to XIST leads to gene silencing remains unclear. In addition to its function as an RNA-binding protein, YTHDC1 promotes H3K9me2 demethylation and gene expression by interacting with and recruiting KDM3B to chromatin regions^51^. The molecular role of the last member of the YTH family, YTHDC2, in the regulation of m6A remains uncertain. YTHDC2 was first found to be an RNA helicase with a YTH domain^52^. Similar to other YTH family proteins, YTHDC2 is able to recognize and bind m6A moieties in mRNA to have a regulatory role^53,54^. Several studies have revealed the importance of YTHDC2 in the meiosis of germline cells. YTHDC2 interacts with MEIOC, a meiosis-specific protein, and XRN1, a 5′–3′ exoribonuclease, to regulate the switch from mitosis to meiosis through posttranscriptional regulation of target transcripts^54–58^. YTHDC2 enhances the translation efficiency of its targets and decreases their mRNA abundance^53^. It is an RNA-induced ATPase with 3′–5′ RNA helicase activity mediated by its DEAH helicase domain^54^. The ankyrin repeats help YTHDC2 to interact with XRN1, UPF1, and MOV10, probably to destabilize its target RNAs^53,54,59^. The binding of YTHDC2 to the 18S rRNA of the small ribosomal subunit in close proximity to the mRNA entry tunnel and exit site suggests that YTHDC2 directly bridges interactions between m6A-containing mRNAs and the ribosome to promote translation^59^. It has also been implied that the R3H domain contributes to RNA binding by stabilizing the interaction between YTHDC2 and its m6A-containing substrates^59^. Interestingly, m6A residues located within the coding sequence (CDS) positively regulate translation by resolving mRNA secondary structures, for which YTHDC2 is required because of its helicase activity^60^. Recently, two hepatic studies also provided evidence that YTHDC2 helps decrease the stability of its target mRNAs and inhibit gene expression in an m6A-dependent manner^61,62^.

## Structural basis for the selective binding of m6A-modified RNA by YTH family proteins

After YT521-B was identified as an RNA splicing-related protein, a conserved region in its protein sequence that is present only in eukaryotic genomes was identified and termed the YT homology (YTH) domain^63^. The YTH domain has been identified in 174 different proteins of eukaryotic species and is abundant in plants. The YTH domain contains 100–150 amino acids and is characterized by 14 invariant and 19 highly conserved residues^64^. In addition to humans, the YTH domain has also been found in Drosophila^63^, fission yeast^65^, *Saccharomyces cerevisiae*^66^, *Plasmodium falciparum*^67^, and many species of plants^68–70^. YTH domain-containing proteins in other eukaryotic species perform many of the same or similar functions as those found in humans. For example, the m6A mRNA methylation program has been revealed in the malaria parasite, and PfYTH1 and PfYTH2, YTH domain-containing proteins in *P. falciparum*, were confirmed to be m6A readers^67,71^. In Arabidopsis, m6A recognition by the YTH domain-containing proteins ECT2, ECT3, and ECT4 are important for cell proliferation and plant organogenesis^72,73^. In fission yeast, Mmi1, a deeply researched YTH domain-containing protein, selectively recognizes a *cis*-acting region (DSR) specific for meiotic transcripts and directs them to the nuclear exosome for degradation^65,74^. Mmi1 also directs RNAi-dependent heterochromatin formation and gene silencing through recruitment of Red1, the histone H3K9 methyltransferase Clr4/SUV39h, the RNA-induced transcriptional silencing (RITS) RNAi complex, and the conserved cleavage and polyadenylation factor (CPF)^75–78^. The role of Mmi1 in affecting chromatin accessibility and heterochromatin is similar to that of its human homolog YTHDC1.

**Fig. 2:**
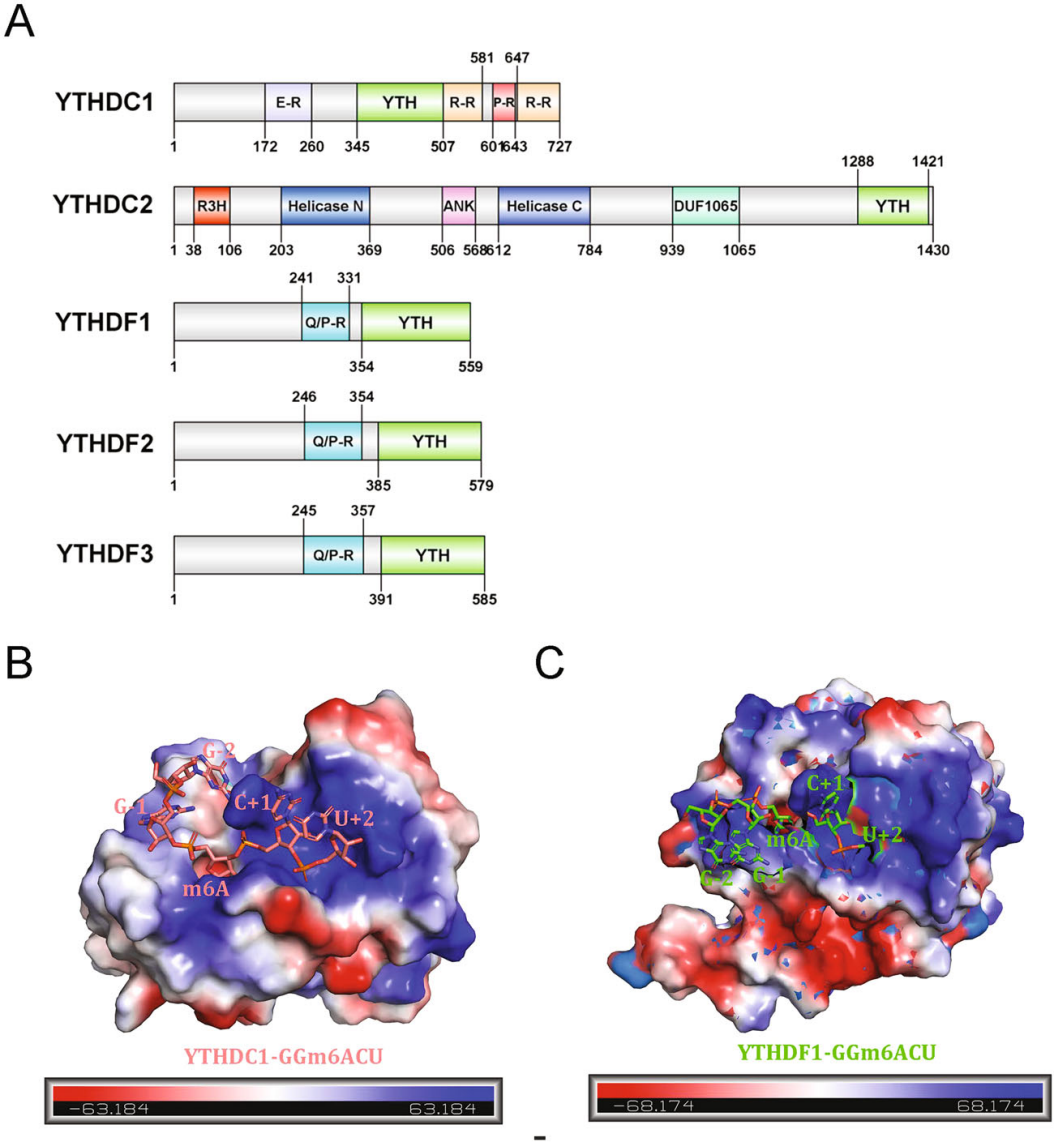
The YTH domain is usually located in the C terminus of proteins and is rich in basic amino acid residues for RNA binding. **A** Schematic representation of the protein structure of human YTH domain-containing proteins (YTHDC1, YTHDC2, YTHDF1, YTHDF2, and YTHDF3). **B, C** The electrostatic potential surface of the YTH domains of YTHDC1 and YTHDF1 in complex with m6A-containing oligonucleotides is represented by PyMOL 2.0. Positive charges are colored blue, neutral charges are white, and negative charges are red.

The presence of a YTH domain defines a group of proteins that includes YTHDF1-3, YTHDC1, and YTHDC2 in humans (Fig. 2A). After the YTH domain was first found to be able to bind single-stranded RNA, these five proteins were successively identified as m6A readers^4,18,53,64,79^. Through NMR spectroscopy and X-ray diffraction analysis, the solution and crystal structures of the YTH domains of distinct YTH family members and their complexes with m6A RNA oligoribonucleotides have been solved successively^80,81^. Overall, these YTH domains commonly adopt a specific mixed α-helix-β-sheet fold in which the β-sheets are arranged into a central, globular β-barrel fold and surrounded by α-helices, providing a hydrophobic core where several highly conserved aromatic residues are located. Electrostatic potential analysis of the protein surface demonstrated that the surface of the YTH domain has a positively charged concave structure, which is enriched with basic residues such as lysines and arginines and is responsible for RNA binding. Intriguingly, a well-defined conserved aromatic cage is observed in all YTH domains, which endows the YTH domain with the capacity for discriminative recognition of N6-methyladenosine-modified RNA (Fig. 2B).

**Fig. 3:**
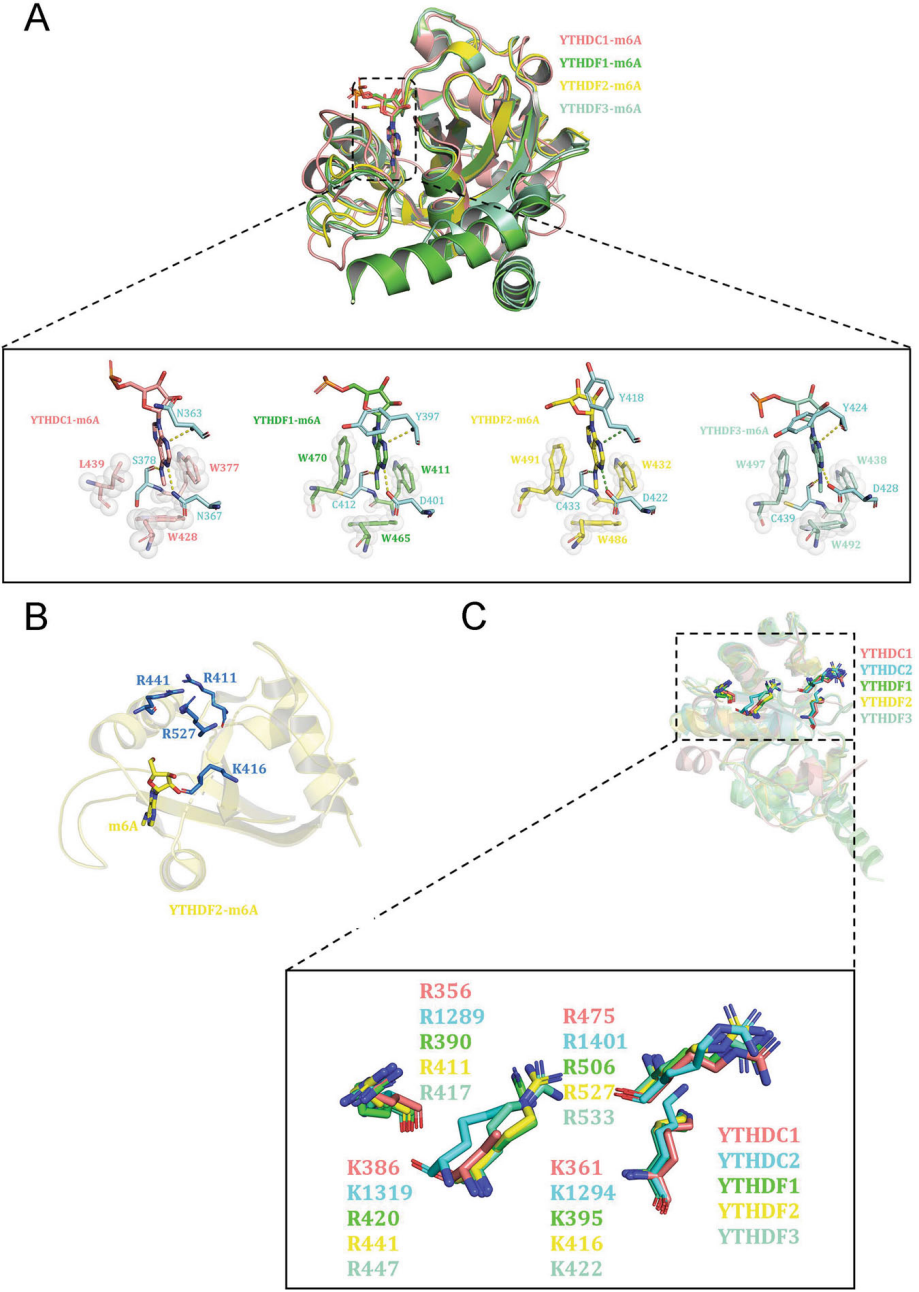
The conserved aromatic residues and basic residues in the YTH domain are responsible for methylated adenosine (m6A) recognition and binding, respectively. **A** Superposition of the complex structures of the YTHDC1 YTH domain (YTH-YTHDC1)-m6A (pink), YTH-YTHDF1-m6A (green), YTH-YTHDF2-m6A (yellow), and YTH-YTHDF3-m6A (pale green), showing the conserved aromatic cage for specific recognition of the methyl group of m6A. Both the residues in the aromatic cage of YTHDC1 and the 6-methylated adenine base (m6A) are shown in the model as pink sticks, and the counterparts in YTHDF1, YTHDF2, and YTHDF3 are shown in the model as green, yellow, and pale green sticks, respectively. In addition, the adenine base always forms three hydrogen bonds with adjacent residues (cyan sticks), which is essential for the binding between m6A and YTH domains. The hydrogen bonds are labeled with dashed lines. The corresponding PDB IDs are 4R3I, 4RCJ, 4RDN, and 6ZOT. **B** Four basic residues, R411, K416, R441, and R527, on the surface of the YTHDF2 YTH domain, are critical for binding to the RNA backbone. **C** Superposition of the YTH domains of YTHDC1 (pink cartoon), YTHDC2 (cyan cartoon), YTHDF1 (green cartoon), YTHDF2 (yellow cartoon), and YTHDF3 (pale green cartoon). The four basic residues are highly conserved in these human YTH proteins. The basic residues R411, K416, R441, and R527 in YTHDF2 and their corresponding residues in the YTH domains of YTHDC1, YTHDC2, YTHDF1, and YTHDF3 are shown as yellow, pink, cyan, green, and pale green sticks.

The first aromatic cage was discovered in the YTH domain of YTHDC1 and consists of two aromatic tryptophans (W377 and W428) and an atypical nonaromatic leucine (L439), which forms vital π-π interactions with the adenine base and hydrophobic interactions with the N6-methyl moiety^79^ (Fig. 3A). Mutation of either W377 or W428 abolishes the binding of YTHDC1 to m6A, highlighting its critical role in specifically recognizing N6-methylated adenine bases. In addition to the above fundamental hydrophobic interactions, the N atoms in the methyladenine base (N6, N3, and N1) form hydrogen bonds with three residues (S378, N363, and N367) of YTHDC1 adjacent to the cage, making the binding between YTHDC1 and m6A more stable (Fig. 3A). Interestingly, Xu et al. found that when N367 is mutated to D367, m6A binding of YTHDC1 is abolished. Considering the difference in the protonation state between N367 and D367, it is predicted that the N1 atom is in a deprotonated state and thus would preferentially bind to deprotonated N367 over protonated D367^82^. In addition, the detailed structural analysis further specified that the basic residues around the aromatic cage also make significant contributions to the binding affinity of YTHDC1 for m6A-modified RNA. For example, the cytidine in GG(m6A)CU not only forms one hydrogen bond with the side chain of R475 but is also stacked with the guanidinium group of R475 and the adjacent uracil through cation–π and π–π interactions, respectively. Mutating R475 to phenylalanine or an alanine reduces the binding affinity by 9-fold or over 100-fold, respectively, suggesting the importance of this residue in maintaining YTHDC1-RNA binding. Recently, the crystal structure of the YTHDC2 YTH domain was solved and compared with that of the YTHDC1 domain in complex with GG(m6A)CU oligonucleotides^83^. Similarly, the YTH domain of YTHDC2 contains three aromatic residues (W1310, W1360, and L1365) corresponding respectively to W377, W428, and L439 of YTHDC1, which constitute a conserved hydrophobic pocket for m6A recognition. Furthermore, a positively charged surface around the m6A binding pocket is also observed in the YTHDC2 YTH domain, indicating that YTHDC2 adopts an architecture similar to that of YTHDC1 to accommodate m6A RNA moieties.

With the clarification of the YTHDC1–m6A complex structure, the structures of three cytoplasmic m6A readers, YTHDF1, YTHDF2, and YTHDF3, in complex with m6A-modified RNA have successively been solved by three individual groups^40,82,84^. Unsurprisingly, the YTH domains of YTHDF1, YTHDF2, and YTHDF3 harbor a specific m6A RNA-binding surface and aromatic cage similar to that of YTHDC1; this cage is composed of W411, W465, and W470 in YTHDF1; W432, W486, and W491 in YTHDF2; and W438, W492, and W497 in YTHDF3 (Fig. 3A). Mutating the above tryptophans to alanines drastically impairs the binding of YTHDF1 and YTHDF2 to m6A RNA. Additionally, three conserved residues in YTHDF1 (C412, Y397, and D401), YTHDF2 (C433, Y418, and D422), and YTHDF3 (C439, Y424, and D428) that are located near the aromatic cage and correspond to S378, N363, and N367 in YTHDC1 form hydrogen bonds with the N6 amino group in m6A, N3 in purine rings, and N1 in purine rings, respectively (Fig. 3A). Mutation of Y397 in YTHDF1 results in a marked decline in its m6A RNA binding, suggesting that these hydrogen bonds are also of great importance in m6A recognition. Of particular note is that N367 in YTHDC1 is substituted for D401 in YTHDF1, which may explain why the binding affinity of YTHDC1 for GG(m6A)CU (*K*_d_ = 2.0 μM) is ten times higher than that of YTHDF1 (*K*_d_ = 22.0 μM)^82^. Mutating D401 to N401 significantly enhances the binding capacity of YTHDF1, rendering it identical to that of YTHDC1 (*K*_d_ = 1.5 μM), confirming the considerable contribution of this specific residue to m6A RNA binding. Moreover, a basic patch composed of four basic residues, R411, K416, R441, and R527, is observed on the electrostatic potential surface of the YTHDF2 YTH domain^85^ (Fig. 3B). Sequence alignment of the five YTH domains showed that these four residues are almost completely conserved in YTHDF1-3 and YTHDC1-2 (Fig. 3C). The results of mutagenesis experiments demonstrated that mutating K416 and R527 of YTHDF2 to alanines not only heavily reduces the m6A binding affinity of YTHDF2 by approximately 25-fold but also decreases the binding of unmethylated RNA (A-RNA) by over 5-fold and 10-fold, respectively. Similarly, the binding affinity of the YTHDF2 R411A mutant for m6A RNA and A-RNA is decreased by ~3-fold and 2-fold, respectively, revealing that the basic patch on the surface of the YTH domain has a dominant role in binding to the RNA backbone but probably does not participate in m6A recognition. To more clearly delineate the molecular mechanisms underlying the specific recognition of m6A-modified RNA by human YTH family proteins, a summary of binding affinities of the wild-type and YTH domain mutants of YTHDC1, YTHDF1, and YTHDF2 to 5-mer and 17-mer m6A-modified RNA oligonucleotides are provided in Table 1.

**Table 1:** Binding affinities of the wild-type and mutant YTH domains of YTHDC1, YTHDF1, and YTHDF2 for m6A-modified RNAs.

RNA sequence	Proteins	Genotype	*K*_d_ (μM)
GG(m6A)CU	YTHDC1	WT	2.0 ± 0.1
		W377A	NB
		W428A	NB
		R475F	18 ± 2
		R475A	210 ± 20
		N376D	>100
GG(m6A)CU	YTHDF1	WT	22 ± 4
		W411A	NB
		W465A	NB
		W470A	NB
		D401N	1.5 ± 0.1
		Y397A	NB
		R506A	>200
UUCUUCUGU	YTHDF2	WT	2.54
GG(m6A)CUGUG		W432A	16.64
		W486A	17.61
		K416A	62.75
		R527A	62.94
		R411A	7.81
		R441A	7.35

*NB* no binding.

**Fig. 4:**
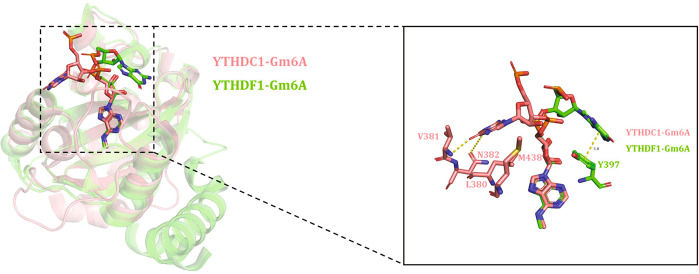
Structural comparison of YTHDC1 and YTHDF1 reveals the structural basis for the discriminative recognition of the nucleotide preceding the m6A mark by YTHDC1. The YTHDC1-G(m6A) and YTHDF1-G(m6A) complexes are shown in the model as pink and green sticks, and the difference interactions between YTHDC1 and YTHDF1 with the G-1 nucleotide are highlighted with yellow dashed lines.

A previous study of the m6A RNA methylome by borate gel chromatography suggested that m6A modification is highly enriched in RRACU (where R is A or G) sequences in mammals^86^. Recent PAR-CLIP studies on transcriptome-wide YTHDC1 binding sites also identified GG(m6A)C as the highest affinity binding motif of YTHDC1^79^. Furthermore, quantitative isothermal titration calorimetry (ITC) binding assay confirmed that the binding affinity of YTHDC1 for GG(m6A)CU (*K*_d_ = 2.0 μM) was severely impaired when the second G nucleotide (G-1) was replaced with A (A-1, *K*_d_ =15.0 μM). However, subsequent comprehensive analysis of the sequence preference of different YTH domains for m6A RNAs revealed that no YTH domain except for that in YTHDC1 exhibits sequence selectivity at the position preceding the m6A modification^82^ (Table 2). By superposition of the crystal structures of the YTHDF1-GG(m6A)CU and YTHDC1-GG(m6A)CU complexes, several key structural divergences were observed that may reasonably explain the unique nucleotide selectivity of YTHDC1 at the -1 position. First, G-1 (the G nucleotide preceding the m6A) forms two hydrogen bonds with V382 and N383 and interacts with L380 and M438 by hydrophobic interactions (Fig. 4), which might be abolished by replacing G-1 with A-1. Second, L380 and M438 are exclusive to YTHDC1, while their counterparts in other YTH proteins are polar amino acids, for example, T414 and K469 in YTHDF1 and T435 and K490 in YTHDF2. Mutating either of these two residues to an alanine not only impairs binding to m6A-containing 16-mer RNA oligonucleotides but also abolishes the sequence preference of YTHDC1 at the −1 position. In contrast, the G-1 nucleotide only forms one hydrophobic interaction with Y397 in YTHDF1, which may result in decreased selectivity between YTHDF1 and the G-1 nucleotide (Fig. 4). In summary, compared to other YTH domains, the YTHDC1 YTH domain adopts a unique G-1 binding pocket, by which YTHDC1 acquires selectivity for the nucleotide preceding the m6A.

**Table 2:** Binding affinities of four human YTH family members (YTHDC1, YTHDC2, YTHDF1, and YTHDF2) to a 9-mer methylated RNA oligonucleotide.

RNA sequence	*K*_d_ (μM)			
	YTHDC1	YTHDC2	YTHDF1	YTHDF2
CCG**A**(m6A)CUGU	1.0 ± 0.1	24 ± 2	1.1 ± 0.2	0.9 ± 0.2
CCG**G**(m6A)CUGU	0.22 ± 0.03	12 ± 2	0.8 ± 0.3	0.9 ± 0.1
CCG**C**(m6A)CUGU	0.32 ± 0.03	14 ± 1	0.8 ± 0.2	0.7 ± 0.2
CCG**U**(m6A)CUGU	0.30 ± 0.06	16 ± 3	0.9 ± 0.2	0.8 ± 0.2

The nucleotide preceding the m6A mark is highlighted in bold to depict the binding preference of the four YTH proteins for different m6A-containing RNAs.

## YTH family proteins’ roles in human cancer

Since N6-methyladenosine modification affects gene expression during multiple steps of RNA metabolic processes, many studies have found that m6A is critical in many diseases, including cancer^87,88^. As important readers, YTH family proteins are involved in almost every aspect of tumorigenesis and cancer progression (Table 3).

**Table 3:** List of the roles of YTH family proteins in different cancers.

Cancer type or procedure	Reader	Main targets	Role
Colorectal cancer	YTHDF1	TCF4	Oncogene
	YTHDF3	GAS5	Oncogene
Hepatocellular carcinoma	YTHDF2	IL11, SERPINE2, EGFR	Tumor suppressor
	YTHDF2	OCT4	Oncogene
	YTHDF2	SOCS2	Mediator
Pancreatic cancer	YTHDF2	YAP signaling	Migration–proliferation dichotomy
Lung cancer	YTHDF1	CDK2, CDK4, cyclin D1, YAP	Oncogene
	YTHDF2	6PGD	Oncogene
	YTHDF3	YAP	Hub of YTHDF1-2
	YTHDC2	SLC7A11	Tumor suppressor
Gastric cancer	YTHDF1	FZD7	Oncogene
Ovarian cancer	YTHDF1	EIF3C	Oncogene
Bladder cancer	YTHDF1	ITGA6, CDCP1	Oncogene
Prostate cancer	YTHDF2	LHPP, NKX3-1	Oncogene
Merkel cell carcinoma	YTHDF1	Unknown	Oncogene
Acute myeloid leukemia	YTHDF2	Tnfrsf2	Oncogene
Epithelial–mesenchymal transition	YTHDF1	Snail	Oncogene
Antitumour immune response	YTHDF1	Lysosomal proteases	Weaken antitumor response
Ocular melanoma	YTHDF1	HINT2	Tumor suppressor
Glioblastoma	YTHDF2	MYC, VEGFA, LXRα, HIVEP2	Oncogene
	YTHDC1	SRSF	Mediator
Endometrial carcinoma	YTHDC1	VEGF, BRCA1, PGR	Oncogene
Cancer metastasis	YTHDF3	ST6GALNAC5, GJA1, and EGFR	Oncogene
	YTHDC2	HIF-1α	Oncogene
Nasopharyngeal carcinoma	YTHDC2	IGF1R	Oncogene

### YTHDF1’s role in human cancer

As mentioned above, YTHDF1, a reader of m6A, can promote the translation of some transcripts to change the proteome in cancer cells, therefore regulating tumorigenesis. Additionally, several kinds of tumors have been reported to be related to YTHDF1. Many studies have indicated that YTHDF1 is an oncogene. For example, YTHDF1 is highly expressed in intestinal stem cells and colorectal cancer cells, and it participates in Wnt signaling. YTHDF1 can facilitate the translation of Wnt signaling effectors, including TCF7L2/TCF4, to augment β-catenin activity to regulate intestinal stem cell activity and tumorigenesis^89,90^. Similarly, in gastric cancer, mutated YTHDF1 enhances the translation of FZD7 to activate the Wnt-β-catenin pathway to promote gastric cancer cell proliferation and tumorigenesis^91^. In non-small cell lung cancer (NSCLC), YTHDF1 was reported to promote cancer cell proliferation and tumor progression by regulating the translational efficiency of CDK2, CDK4, and cyclin D1^92^. In addition, YTHDF1 helps promote YAP mRNA translation in NSCLC, and the increases in YAP expression and activity induce drug resistance and metastasis in NSCLC^93^. Abnormally controlled translation of key mRNAs in the cancer genome and generally enhanced translational output are important responses to oncogenic stimulation^94^. Indeed, in ovarian cancer, m6A-modified EIF3C, which is an essential initiation factor, is recognized and bound to YTHDF1. YTHDF1 promotes the translation of EIF3C and therefore enhances the total translational output, inducing cancer progression and metastasis^95^. It was also reported that YTHDF1 has an important role in bladder cancer. YTHDF1 helps promote the translation of ITGA6 and CDCP1 mRNA, and high expression of these factors can increase the growth and progression of bladder cancer^96,97^. Merkel cell carcinoma is deadly skin cancer in which YTHDF1 was found to be highly expressed and associated with tumorigenesis^98^. Additionally, YTHDF1 has been implicated in epithelial–mesenchymal transition (EMT), of which the transcription factor Snail is known to be a critical regulator. YTHDF1 was reported to mediate the m6A-increased translation of Snail mRNA and thus regulate EMT in cancer cells^19^. In the field of tumor immunotherapy, it was reported that durable neoantigen-specific immunity is suppressed by YTHDF1. Mechanistically, m6A-containing transcripts encoding lysosomal proteases are recognized by YTHDF1, and thus elevated expression at the translational level promotes the degradation of tumor neoantigens and represses cross-presentation to influence the efficacy of immunotherapy^99^. Moreover, there is some evidence indicating that YTHDF1 is associated with poor prognosis in patients with hepatocellular carcinoma and breast cancer^100–103^. However, YTHDF1 was also found to act as a tumor suppressor by promoting the translation of the methylated mRNA of HINT2, a tumor suppressor in ocular melanoma^104^.

### YTHDF2’s role in human cancer

Another important reader, YTHDF2, is also well researched and has been found to be closely related to human cancer. Among the many different kinds of tumors, hepatocellular carcinoma (HCC) is one in which YTHDF2 has been studied the most. YTHDF2 can influence tumor progression in several different ways. YTHDF2 generally works as a tumor suppressor in HCC, as it mediates the decay of m6A-containing IL11 and SERPINE2 mRNAs, which are mediators of cancer-promoting inflammation and reprogramming of the tumor vasculature^69^. YTHDF2 also suppresses ERK/MAPK signaling by destabilizing EGFR in an m6A-dependent manner to inhibit the growth and proliferation of HCC cells^105^. Moreover, YTHDF2 participates in HCC progression in another way, although it does not have a core role. It binds SOCS2 mRNA and mediates its degradation to promote liver cancer progression, while METTL3 regulates the m6A level of SOCS2 mRNA^106^. It was reported that miR-145 targets the 3′ UTR of YTHDF2 mRNA, thus affecting the decay of m6A-containing mRNA to influence the m6A level in HCC cells. However, this research showed that YTHDF2 was closely associated with the malignancy of HCC^107^. Indeed, another research group regarded YTHDF2 as an oncogene in HCC because they found that YTHDF2 increased OCT4 expression to promote liver cancer metastasis^108,109^. Regarding other types of cancer, YTHDF2 is overexpressed in acute myeloid leukemia and is required for disease initiation. Mechanistically, YTHDF2 destabilizes m6A-modified transcripts such as that of the tumor necrosis factor receptor Tnfrsf2 to protect self-renewing leukemic stem cells against apoptosis^110^. YTHDF2 promotes the proliferation and inhibits the migration and invasion as well as EMT of pancreatic cancer cells, probably through YAP signaling, although the exact mechanism remains to be clarified^111^. The oncogenicity of YTHDF2 was revealed in prostate cancer, and YTHDF2 mediates the degradation of LHPP and NKX3-1 to induce the phosphorylation of AKT^112^. In glioblastoma, in contrast to its proposed role, YTHDF2 was shown to stabilize MYC and VEGFA to maintain the oncogenic phenotype of glioblastoma stem cells^113^. It was also reported that YTHDF2 was phosphorylated at serine 39 and threonine 381 through EGFR/SRC/ERK signaling in glioblastoma^114^. The phosphorylation of YTHDF2 stabilized the YTHDF2 protein and promoted the decay of LXRA and HIVEP2 mRNA, which is required for cholesterol dysregulation, cell proliferation, invasion, and tumorigenesis in glioblastoma. Interestingly, although YTHDF2 was regarded to control mRNA decay, YTHDF2 was reported to facilitate 6PGD mRNA translation to promote lung cancer cell growth^115^, similar to the role of YTHDF1 and the same effect as the increased OCT4 expression mentioned above^108^. Notably, YTHDF2 has an important role in the regulatory effects of m6A methylases and demethylases on the tumorigenicity of osteosarcoma, breast tumors, melanoma, bladder cancer, pancreatic cancer, and colorectal cancer^116–123^.

### Other readers in human cancer

The other three YTH family proteins, YTHDF3, YTHDC1, and YTHDC2, were found to be less correlated with human cancer than YTHDF1 and YTHDF2. Recently, YTHDF3 was found to be overexpressed and enhance the translation of ST6GALNAC5, GJA1, and EGFR to promote brain metastasis in breast cancer^109^. A negative functional loop constituted by the lncRNA GAS5-YAP-YTHDF3 axis was revealed in colorectal cancer, as GAS5 interacts with the WW domain of YAP to facilitate YAP shuttling from the nucleus to the cytoplasm and YAP phosphorylation; subsequently, YAP is degraded in a ubiquitin-mediated manner to inhibit CRC progression. Importantly, YTHDF3 is a target of YAP signaling and mediates the decay of m6A-modified GAS5 mRNA^124^. Regarding YTHDF3, research in NSCLC revealed the mechanism by which YTHDF3 acts as a hub to fine-tune the accessibility of RNA to YTHDF1 and YTHDF2^30^. As demonstrated above, YAP mRNA is recognized by YTHDF3 and is then assigned to YTHDF1 or YTHDF2 to be destabilized or translated; therefore, YTHDF3 is able to control YAP signaling to regulate cell proliferation, metastasis, and other tumorigenic behaviors. In addition, YTHDC1, which is an m6A reader involved in RNA splicing, was reported to recognize m6A modification around the start codon of serine/arginine-rich splicing factors (SRSFs) and lead to nonsense-mediated mRNA decay, which affects the alternative splicing of a number of genes, such as BCL-X and NCOR2, eventually causing cancer-related phenotypes mediated by METTL3 in glioblastoma^125^. In addition, the ability of YTHDC1 to splice transcripts has been demonstrated for a vascular endothelial growth factor (VEGF), breast cancer 1 (BRCA1), and the progesterone receptor (PGR) in endometrial carcinoma, but whether these processes are dependent on m6A modification is still unclear^126,127^. YTHDC2, the last reader, was reported to be upregulated in human cancer cell lines and to promote cancer metastasis by promoting translation initiation via unwinding of the 5′-untranslated region (5′ UTR) of mRNAs such as HIF-1α^128^. Moreover, YTHDC2 was found to be highly expressed in radioresistant nasopharyngeal carcinoma and to promote radioresistance by activating the IGF1R/ATK/S6 signaling axis^129^. YTHDC2 also regulates redox homeostasis and inhibits LUAD tumorigenesis since it promotes m6A-dependent mRNA degradation of SLC7A11, which is the core component of a cystine/glutamate antiporter^130^.

## Conclusions and perspectives

In the early years, studies about the association between m6A modification and human cancer were centered around the balance of m6A addition and removal by writers and erasers, respectively^6,131–133^. However, changes in the readers could be a more crucial factor in the fate of RNAs. Therefore, we ask whether the switch from one reader to another could be a more reasonable strategy to control gene expression. Indeed, readers have recently been increasingly emphasized in epigenetic m6A modifications. Since domains other than the YTH domain of YTHDFs may only function after the binding process, it is likely that the YTH domain is responsible for recognizing target mRNAs. However, the targets of different readers have a certain degree of overlap, and the mechanism underlying the selectivity of readers is not well understood and may involve preferred motifs, phase separation, or the possible assigning function of YTHDF3 and other unknown factors. The upstream regulation of YTH family proteins is still unclear, although it has been found that miR-145 may affect YTHDF2 mRNA^107^. It is worth noting that there is an emerging concept that YTHDF proteins are redundant in function and that their only effect is destabilizing transcripts, which makes this field more complicated. In different types of tumor cells, the specific regulatory mechanism of m6A differs. It is possible that the functions of different readers can partially overlap, and this possibility still needs to be further investigated. From the perspectives of translational medicine and clinical medicine, drugs targeting the YTH family may be a potential strategy for certain cancers. Although specific chemical inhibitors targeting the YTH domain have yet to be discovered, the above investigation of the structural biology of different YTH domains has paved the way for the rational design of small-molecule YTH domain inhibitors. Notably, through a recent virtual screen and crystallographic analysis, Rajiv et al. identified some promising hits as competitive YTH domain modulators that were expected to efficiently disrupt the interactions between m6A and YTHDC1. Specifically, the author indicated that N-methyl amides could constitute appropriate fragments to compete with m6A molecules. Overall, considering the importance of the YTH family in cancer progression, the development of specific YTH domain inhibitors would not only enhance our knowledge of cancer epigenetics but also provide novel targeted therapies.
